# Genetic Risk Scores and Missing Heritability in Ovarian Cancer

**DOI:** 10.3390/genes14030762

**Published:** 2023-03-21

**Authors:** Yasaman Fatapour, James P. Brody

**Affiliations:** Department of Biomedical Engineering, University of California, Irvine, CA 92697, USA

**Keywords:** copy number variation, ovarian cancer, machine learning, germline, UK Biobank, TCGA

## Abstract

Ovarian cancers are curable by surgical resection when discovered early. Unfortunately, most ovarian cancers are diagnosed in the later stages. One strategy to identify early ovarian tumors is to screen women who have the highest risk. This opinion article summarizes the accuracy of different methods used to assess the risk of developing ovarian cancer, including family history, BRCA genetic tests, and polygenic risk scores. The accuracy of these is compared to the maximum theoretical accuracy, revealing a substantial gap. We suggest that this gap, or missing heritability, could be caused by epistatic interactions between genes. An alternative approach to computing genetic risk scores, using chromosomal-scale length variation should incorporate epistatic interactions. Future research in this area should focus on this and other alternative methods of characterizing genomes.

## 1. Background

Ovarian cancer is known as the silent killer. The symptoms of ovarian cancer in the initial stages are minimal and non-specific. Constipation, heartburn, fatigue, and bloating are early signs of ovarian cancer, but they are also associated with other common maladies. Because of these non-specific symptoms, ovarian cancer is often undiagnosed until the tumor has grown large, spread to nearby organs, and invaded the lymph system. At these later stages, treatment options are limited, and so is survival time. Ovarian tumors, like most solid tumors, can be surgically removed if found early. Removal of the tumor often leads to a complete recovery [1]. However, most early detection strategies for ovarian cancer are ineffective for screening average risk women [2].

Current risk assessment tools for ovarian cancer do not work well enough. Specific genetic tests on BRCA1/BRCA2 status are available and work well for ovarian cancer, but only a small fraction (about 10%) of ovarian cancers are associated with those variants [3]. Otherwise, risk assessment is usually based on family history, but many people have limited knowledge of their family history and in any case a germline genetic test should work better than a perfect family history. Development of a genetic test to identify women at high-risk of ovarian cancer could lead to a reduction in the number of ovarian cancer deaths.

Genetic mutations are known to cause ovarian cancer, but the full extent is not known. The *BRCA1/2* mutations account for a small percentage of ovarian cancer, but the others are called sporadic, with no known genetic cause. We suggest that these sporadic ovarian cancers are caused by as of yet unknown genetic alterations in the germline. These genetic alterations might consist of epistatic effects, multiple combinations of mutations, unlike the simple mutations present in *BRCA1/2* that cause ovarian cancer. This suggestion is based on several lines of evidence. First, analysis of somatic mutational data from tumors suggests that these tumors take decades to develop [4]. Second, age specific incidence data suggest that most cancers originate during development [5]. Third, the current lack of a detailed search for epistasis in germlines of ovarian cancer patients. Finally, an alternative hypothesis that cancers originate from exposure to environmental mutagens that cause point mutations in people is not supported by evidence despite decades of studies [6].

## 2. Quantifying the Accuracy of Predictive Tests

Predictive tests often produce a numerical score that can be a continuous value, for instance from 1–100. From this score, one has to choose a cutoff value to make a prediction, which is a binary choice. Parameters like the sensitivity, specificity, positive predictive value, and negative predictive value are all a function of both the test and the choice of a cutoff value. The best way to characterize such a predictive test is with a Receiver Operating Characteristic (ROC) curve [7,8]. This curve represents all cut off values, and one can read the sensitivity and specificity for the test for a given cutoff value.

The AUC (area under the receiver operating characteristic curve) characterizes different predictive tests. The AUC, sometimes called a c-statistic, reduces the ROC curve to a single number, which is useful for comparing different tests. However, the complete ROC curve can show that two tests with similar AUC are not equivalent in some instances. Thus, it is always best to examine the ROC curve for a test when judging its effectiveness.

The AUC can vary from 0.5, which is equivalent to random guessing, to 1.0, which indicates a perfect test that is always correct. The AUC is equivalent to the accuracy when the two classes have equal numbers. The AUC is insensitive to class imbalance.

One example that illustrates how a predictive test with a low AUC can still be effective is the BRCA1 test for breast and ovarian cancer. This test works very well but only in a small subpopulation. Although the AUC is small, the test is quite valuable for that subpopulation.

## 3. Theoretical Maximum Accuracy of an Ovarian Cancer Genetic Risk Score

The highest possible AUC for predicting ovarian cancer in women is about 0.99 [9]. The discriminative accuracy of a genetic test depends on two factors, the heritability and prevalence of the trait. The Nordic Twin Study measured the heritability of ovarian cancer at about 40% [10]. Based on this heritability measurement and the prevalence of ovarian cancer, an ovarian cancer genetic test could have a maximum discrimination accuracy (AUC) in excess of 0.99. A substantial gap exists between the current best genetic risk tests and what should be possible.

## 4. Predicting Risk: Family History

Understanding a patient’s family history is the first step in predicting whether a woman will develop ovarian cancer. Predictions based solely on family history have not been well characterized for ovarian cancer, but breast cancer predictive models have been well characterized, as it occurs ten times more frequently than ovarian cancer. For instance, one commonly used predictive model, the Gail model [11], has an AUC of 0.58 (95% confidence interval [CI] = 0.56 to 0.60) [12]. The Gail model incorporates several parameters including first degree relatives who were diagnosed with breast cancer but does not include any genetic information. Certain germline mutations in *BRCA1* and *BRCA2* are known to increase the risk of ovarian cancer.

The Tyrer-Cuzick model includes a more detailed picture of genetics, including *BRCA1/BRCA2* status and a hypothetical low-penetrance gene that is designed to encompass all other genetic factors [13]. The Tyrer-Cuzick model is an improvement over the Gail model and has an AUC = 0.62, with a 95% CI of (0.60 to 0.64) [14].

Several mutations in the *BRCA1/BRCA2* genes are known to increase the risk of developing ovarian cancer. However, these mutations account for only about 10% of ovarian cancers in the general population [3,15]. Similarly, the fraction of breast cancers attributable to mutations in *BRCA1* or *BRCA2* is about 10%. Thus, the best AUC which could be expected for ovarian cancer predictive tests based on family history and supplemented with information on *BRCA1/BRCA2* mutation status is probably similar to that for breast cancer, or about AUC = 0.60–0.65 [16,17,18,19,20,21,22].

The *BRCA1/2* genetic tests are used to predict women at a high risk of breast and ovarian cancers. Some women whose BRCA test indicates a high risk of breast cancer choose to surgically remove their breasts to avoid breast cancer. Although less common, some women also choose a prophylactic oophorectomy—the surgical removal of the ovaries—to avoid ovarian cancers.

A positive *BRCA1/2* test is highly predictive of breast/ovarian cancer, but a negative test is not very predictive of not having these cancers. In the US, only about 5–10% of breast and ovarian cancers are associated with mutations in *BRCA1/2*. A need exists to develop an effective genetic test for these other 90–95% of breast and ovarian cancers.

## 5. Predicting Risk: Polygenic Risk Scores

To fill this need, the most common approach is to use polygenic risk scores [16,17,18,19,20,21,22]. These are linear combinations of single nucleotide polymorphisms (SNPs) found more often in breast/ovarian cancer patients than in the general population. Models based on detailed germline genetics should perform better than models based on family history alone, since family history is often incomplete, limited to just a generation or two, and genetic factors present in relatives might not be inherited.

The polygenic risk scores used today originate from Genome Wide Association Studies (GWAS) [23,24,25]. These GWAS studies were designed to find genes that drive disease, not for predictive tests. These polygenic risk scores are usually computed as a linear combination of the “hits,” each with a different weight, found in GWAS studies. Different algorithms use slightly different criteria to decide on which “hits” to include and how to weigh them.

The current state of research knowledge on ovarian cancer genetic risk scores is best represented by two recent papers. The first was published in the Journal of the National Cancer Institute in 2020 [26] and the second was published in the European Journal of Human Genetics in 2022 [27].

The 2020 paper [26] evaluated polygenic risk scores for ovarian cancer and seven other common cancers using the UK Biobank. In this dataset, they identified 358 women who had been diagnosed with ovarian cancer. They constructed a polygenic risk score based upon 31 different SNPs. Then, they evaluated the performance of this polygenic risk score to predict ovarian cancer using the UK Biobank dataset. This test had a predictive accuracy of AUC = 0.568 (95% CI 0.537 to 0.598).

The second paper, with over 150 authors, is a *tour-de-force* [27]. Compared to the first paper, they increase the number of ovarian cancer subjects by nearly a factor of 100, using 23,564 cases. They thoroughly explored different combinations of SNPs and different algorithms for combining these SNPs into a polygenic risk score. The second paper [27] describes the best model found to be one based on measurements of 27,240 SNPs, almost 1000 times more than the 2020 paper [26]. After all that optimization, they achieved an AUC of 0.588 (they did not report a 95% confidence interval for the AUC).

Comparing the two papers, one can see that despite the extraordinary efforts of the second paper, the AUC of the test was not significantly higher than the first paper (AUC = 0.588 vs. 95% CI 0.537 to 0.598). From this comparison, we can conclude that most of the useful information for predicting ovarian cancer has been extracted from SNP data using current algorithms. It seems unlikely that the AUC can be significantly improved with different algorithms, a different set of SNPs, or more patients in a dataset. This AUC is substantially lower than the theoretical maximum; something is missing.

## 6. Missing Heritability?

Many human diseases, including ovarian cancer, are known to be inherited. It was thought that the advent of large-scale genome wide association studies (GWAS) would reveal the underlying genes that led to this inheritance for different diseases [28,29]. However, GWAS results have consistently shown that a substantial gap exists between the heritability that could be attributed to known factors by GWAS and the heritability observed by studying inheritance in families. The size of this gap varies by disease or trait, but it can be as large as a factor of ten [30]. The general missing heritability problem, and potential solutions, is well described by [29], in the specific case of ovarian cancer; Flaum et al. put it succinctly: “However, a significant proportion of women who develop ovarian cancer with a strong family history of breast and/or ovarian cancer still do not have a known variant to explain their increased risk, and there must be other genetic factors at play that we do not yet understand” [15].

## 7. Beyond Polygenic Risk Scores

Epistatic interactions, nonadditive interactions between two or more genes, are one factor usually cited as part of the missing heritability problem [29,31]. The methods used in GWAS studies ignore non-linear interactions between genes, which are necessary to measure epistatic interactions. Modern statistical techniques, or machine learning, allow one to consider non-linear interactions between features, but these techniques inevitably require substantially more features (SNPs) than samples (patients), which is not useful when a few thousand patient samples are considered large, and genomes are characterized by millions of SNPs.

One approach to the problem is to construct a different representation of the genome as an alternative to SNPs. A more compact representation that still accounts for the variability in humans would allow the use of machine learning algorithms.

**Figure 1:**
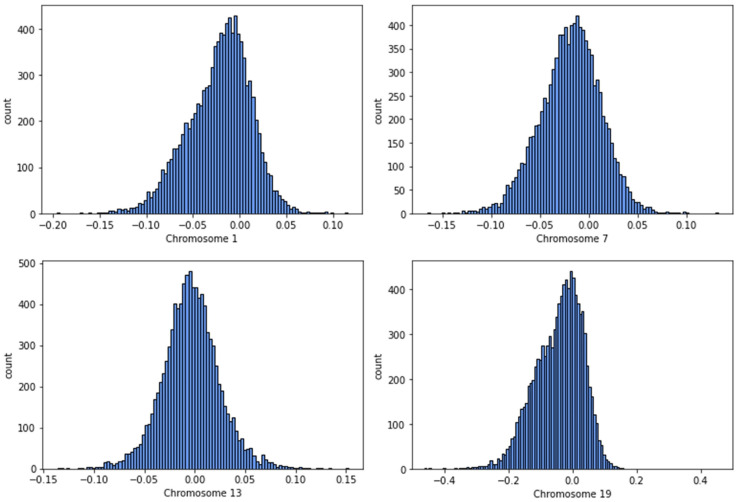
This figure shows a histogram of chromosome-scale length variation measurements of chromosomes 1, 7, 13, and 19 for 10,000 people in the NIH All of Us dataset. “Chromosome length” is measured by averaging calibrated intensity measurements taken from SNP arrays for many SNPs located on each of the four chromosomes. These calibrated intensity measurements are representative of local copy number. Chromosomes can have many deletions, insertions, and translocations that affect copy number. The values measured in log_2(Ratio Units) represent the overall length of the chromosome, where a value of zero indicates the nominal average chromosome length. By measuring this parameter for all chromosomes, one can characterize each person’s germline genetic makeup with these 23 numbers.

One example of this approach is to use measures of chromosome-scale length variation [32]. Chromosome-scale length variation can be computed from SNP array data. SNP arrays provide calibrated intensity values for each SNP location. This intensity data is usually processed into copy number variation data, which is represented by a multiplicity number (where two is the normal multiplicity) and chromosome segment. Instead, one can take this intensity data and compute an average multiplicity across an entire chromosome. By measuring this multiplicity across an entire chromosome for many people, one finds a distribution in values (See Figure 1). A person’s germline genome, then, can be characterized by a series of 23 numbers where each number represents the average multiplicity across each chromosome.

This representation of a person’s genome as 23 numbers has some advantages over the conventional SNP representation of a genome. It is more compact, but still sufficiently complex to capture the enormity of the human population. The compactness allows one to use modern machine learning techniques. It is extensible; you can split the chromosomes into arbitrarily small sections.

Using a dataset acquired as part of the Cancer Genome Atlas (TCGA) project, we evaluated a genetic risk score computed from chromosomal-scale length variation derived from TCGA normal blood samples. In this dataset, the genetic risk score had an AUC of 0.88 (95% CI of 0.86-0.91) [32]. Women with the highest 20% had 160 times greater risk of developing ovarian cancer as compared to the lowest 20%. Although these numbers showed extraordinary discrimination, it is unclear whether these results can be generalized to the general population. The TCGA dataset only contains people who had been diagnosed with cancer, so this work really distinguished one form of cancer from other forms of cancer. It is also possible that the TCGA has subtle batch effects, leading to falsely high discrimination [33,34].

## 8. Conclusions

Ovarian cancer is completely curable in the early stages. While convincing data do not yet exist, we believe that the propensity to develop ovarian cancer appears to be transmitted through the genome, primarily through epistatic interactions. Thus, our opinion is that identification of signatures in the germline genome that indicate future diagnosis of ovarian cancer should be a primary and important target of research. We describe one early effort to use chromosome-scale length variation measurements to quantify insertions and deletions that might hold promise for predicting risk of developing ovarian cancer.

## Data Availability

Data used in this paper for Figure 1 is available for approved users on the controlled tier of the NIH All of Us researcher workbench at https://www.researchallofus.org/data-tools/workbench/.

## References

[1] Siegel R.L., Miller K.D., Fuchs H.E., Jemal A. (2022). Cancer statistics. CA Cancer J. Clin..

[2] Grossman D.C., Curry S.J., Owens D.K., Barry M.J., Davidson K.W., Doubeni C.A., Epling J.W., Kemper A.R., Krist A.H., Kurth A.E. (2018). Screening for ovarian cancer US preventive services task force recommendation statement. JAMA.

[3] Ramus S.J., Gayther S.A. (2009). The Contribution of BRCA1 and BRCA2 to Ovarian Cancer. Mol. Oncol..

[4] Beerenwinkel N., Antal T., Dingli D., Traulsen A., Kinzler K.W., Velculescu V., Vogelstein B., Nowak M.A. (2007). Genetic Progression and the Waiting Time to Cancer. PLoS Comput. Biol..

[5] Brody J.P. (2014). The Age Specific Incidence Anomaly Suggests that Cancers Originate During Development. Biophys. Rev. Lett..

[6] Thilly W.G. (2003). Have environmental mutagens caused oncomutations in people?. Nat. Genet..

[7] Moons K.G.M., Groot J.A.H.D., Linnet K., Reitsma J.B., Bossuyt P.M.M. (2012). Quantifying the Added Value of a Diagnostic Test or Marker. Clin. Chem..

[8] Florkowski C.M. (2008). Sensitivity, specificity, receiver-operating characteristic (ROC) curves and likelihood ratios: Communicating the performance of diagnostic tests. Clin. Biochem. Rev..

[9] Janssens A.C.J.W., Aulchenko Y.S., Elefante S., Borsboom G.J.J.M., Steyerberg E.W., van Duijn C.M. (2006). Predictive testing for complex diseases using multiple genes: Fact or fiction? Genetics in Medicine. Nature.

[10] Mucci L.A., Hjelmborg J.B., Harris J.R., Czene K., Havelick D.J., Scheike T., Graff R.E., Holst K., Möller S., Unger R.H. (2016). Familial Risk and Heritability of Cancer Among Twins in Nordic Countries. JAMA.

[11] Gail M.H., Brinton L.A., Byar D.P., Corle D.K., Green S.B., Schairer C., Mulvihill J.J. (1989). Projecting Individualized Probabilities of Developing Breast Cancer for White Females Who Are Being Examined Annually. Gynecol. Oncol..

[12] Chlebowski R.T., Anderson G.L., Lane D.S., Aragaki A.K., Rohan T., Yasmeen S., Sarto G., Rosenberg C.A., Hubbell F.A. (2007). Predicting risk of breast cancer in postmen-opausal women by hormone receptor status. J. Natl. Cancer. Inst..

[13] Tyrer J., Duffy S.W., Cuzick J. (2004). A breast cancer prediction model incorporating familial and personal risk factors. Stat. Med..

[14] McCarthy A.M., Guan Z., Welch M., Griffin M.E., Sippo D.A., Deng Z., Coopey S.B., Acar A., Semine A., Parmigiani G. (2019). Performance of Breast Cancer Risk-Assessment Models in a Large Mammography Cohort. Gynecol. Oncol..

[15] Flaum N., Crosbie E.J., Edmondson R.J., Smith M.J., Evans D.G. (2020). Epithelial ovarian cancer risk: A review of the current genetic landscape. Clin. Genet..

[16] Torkamani A., Wineinger N.E., Topol E.J. (2018). The personal and clinical utility of polygenic risk scores. Nat. Rev. Genet..

[17] Lewis C.M., Vassos E. (2020). Polygenic risk scores: From research tools to clinical instruments. Genome Med..

[18] Lambert S.A., Abraham G., Inouye M. (2019). Towards clinical utility of polygenic risk scores. Hum. Mol. Genet..

[19] Hughes E., Tshiaba P., Gallagher S., Wagner S., Judkins T., Roa B., Rosenthal E., Domchek S., Garber J., Lancaster J. (2020). Development and Validation of a Clinical Polygenic Risk Score to Predict Breast Cancer Risk. JCO Precis. Oncol..

[20] Khera A.V., Chaffin M., Aragam K.G., Haas M.E., Roselli C., Choi S.H., Natarajan P., Lander E.S., Lubitz S.A., Ellinor P.T. (2018). Genome-wide polygenic scores for common diseases identify individuals with risk equivalent to monogenic mutations. Nat. Genet..

[21] Sugrue L.P., Desikan R.S. (2019). What Are Polygenic Scores and Why Are They Important?. JAMA.

[22] Li R., Zhang X., Li B., Feng Q., Kottyan L., Luo Y., Sawicki K.T., Khan A., Limdi N., Puckelwartz M. (2022). Polygenic risk vectors (PRV) improve genetic risk stratification for cardio-metabolic diseases. medRxiv.

[23] Goode E.L., Chenevix-Trench G., Song H., Ramus S.J., Notaridou M., Lawrenson K., Widschwendter M., Vierkant R.A., Larson M.C., Kjaer S.K. (2010). A genome-wide association study identifies susceptibility loci for ovarian cancer at 2q31 and 8q24. Nat. Genet..

[24] Evans D.M., Visscher P.M., Wray N.R. (2009). Harnessing the information contained within genome-wide association studies to improve individual prediction of complex disease risk. Hum. Mol. Genet..

[25] Reid B.M., Permuth J.B., Chen Y.A., Fridley B.L., Iversen E.S., Chen Z., Jim H., Vierkant R.A., Cunningham J.M., Barnholtz-Sloan J.S. (2019). Genome-wide Analysis of Common Copy Number Variation and Epithelial Ovarian Cancer Risk. Cancer Epidemiol. Biomark. Prev..

[26] Jia G., Lu Y., Wen W., Long J., Liu Y., Tao R., Li B., Denny J.C., Shu X.-O., Zheng W. (2020). Evaluating the Utility of Polygenic Risk Scores in Identifying High-Risk Individuals for Eight Common Cancers. JNCI Cancer Spectr..

[27] Dareng E.O., Tyrer J.P., Barnes D.R., Jones M.R., Yang X., Aben K.K., Adank M.A., Agata S., Andrulis I.L., Anton-Culver H. (2021). Polygenic risk modeling for prediction of epithelial ovarian cancer risk. Eur. J. Hum. Genet..

[28] Manolio T.A., Collins F.S., Cox N.J., Goldstein D.B., Hindorff L.A., Hunter D.J., McCarthy M.I., Ramos E.M., Cardon L.R., Chakravarti A. (2009). Finding the missing heritability of complex dis-eases. Nature.

[29] Eichler E.E., Flint J., Gibson G., Kong A., Leal S.M., Moore J.H., Nadeau J.H. (2010). Missing heritability and strategies for finding the underlying causes of complex disease. Nat. Rev. Genet..

[30] Génin E. (2020). Missing heritability of complex diseases: Case solved?. Hum. Genet..

[31] Zuk O., Hechter E., Sunyaev S.R., Lander E.S. (2012). The mystery of missing heritability: Genetic interactions create phantom heritability. Proc. Natl. Acad. Sci. USA.

[32] Toh C., Brody J.P. (2021). Genetic risk score for ovarian cancer based on chromosomal-scale length variation. BioData Min..

[33] Jain S., Mazaheri B., Raviv N., Bruck J. (2019). Short Tandem Repeats Information in TCGA is Statistically Biased by Amplification. bioRxiv.

[34] Jain S., Mazaheri B., Raviv N., Bruck J. (2021). Glioblastoma signature in the DNA of blood-derived cells. PLoS ONE.

